# Catheter and Sound

**Published:** 1834

**Authors:** Anthony Hermange

**Affiliations:** Cincinnati


### VI.—Catheter and Sound.

Dr. Drake,

Dear sir,

I was glad to receive your hint, regarding the use of the words grooved catheter, &c. employed in my essay on lithotomy, in your last number. I confess, I should have reflected, that the subject was intended to be submitted to American judgement, and that, of course, the technicalities should conform to the meaning in vogue among American and English writers and readers.

The word *sound*, from its evident import, imples an instrument intended for determining whether a stone is or is not present in the bladder, in a manner analogous to sounding the bottom of the sea, to ascertain its nature, depth, &c. In this country, we so understand the meaning of the word, and employ the sound when grooved as a director.

The word *catheter* would seem to have a more *vague* derivation from the Greek word, signifying *to plunge*, or *thurst into*, which would equally apply to any instrument made to pass into the urethra, although we are accustomed to confine its signification to hollow tubes, destined for the evacuation of urine, when patients are unable to void it. It would, however, appear, that *according to the strictness of language*, we might make use of the word *catheter* as a *generic term*, including the hollow tubes just mentioned, as well as sounds, bougies, &c. And so it is universally admitted in France, although, at the present day, in that country, it is *almost exclusively* applied to designate the instrument used for the direction of the cutting instruments, &c., in lithotomy.

I went to that country with our national prepossession (if I may say so) for our own understanding of the *svecific mean-*

*ing* of the words *catheter* and *sound*, and with the idea that they were every where so understood; but I soon found that we took *contrary sides* as to their application; and that although the *indiscriminate* use of them was there tolerated, still the word catheter was *almost universally* understood as sound is here, and vice versa. Long habit of confounding terms (at least these two) when listening to French verbal instruction, as well as in French reading, must be offered as an excuse for occasional technical gallicism.

For the benefit of such of the readers of the Journal, as are not aware of the difference of acceptation, I will subjoin a translation of the definitions, as given by J. Cloquet, in the Dictionnarie de Medecine Chirurgie, &c., of these two words as indicative of their present application in France.

"Catheter. (Catheter s. m. *katheter* from *kathienai* to plunge-or thrust into.) An instrument that is introduced into the bladder, to evacuate the urine contained therein, *to ascertain the presence of calculi*, to inject various fluids, *to guide the lithotome in the operation for stone*, and lastly, to dilate the canal of the urethra. Various kinds of catheters have been distinguished;—*solid* and flexible catheters, called bougies; catheters which are *hollow* in their whole length, and which are called algalis, or sounds; lastly, the *word catheter*, appears, at the present day, to be *almost exclusively confined* to the *solid* instrument employed in the operation for stone. This instrument is a *solid*, grooved sound, straight at one of its extremities, which is flattened out for the purpose of holding it more firmly, and having an elliptical curvature towards its other extremity. It is made of steel, that it may invariably preserve its curvature, and, at the same time, to allow the lithotome and bistoury to pass along the grove, without danger of interruption from cutting into the metal, which might happen, were it made of a softer material. The groove extends along its convexity to within three or four lines of its extremity, and should be perfectly hollowed out.

Sound. (Specillum, *mela* of the Latins, *mele* of the Greeks.) A surgical instrument which is introduced into the cavity of certain organs, into the tracks of wounds, of fistulœ, &c., to answer divers therapeutic indications. Also to ascertain the condition of the bladder, to evacuate the urine it contains, and to verify the presence of foreign bodies, &c. Surgeons ordinarily employ *hollow* sounds made of silver, whose length, diameter, form and curvature vary according to the age, or sex of the patient, and the circumstances of each case. They are frequently enough called *algalis*. The back or extremity, by which the sound is introduced, is ordinarily blunt, some times conical, and provided on the sides with two openings called the eyes. The other extremity (*le pavillon*) is more enlarged, and has two rings attached to it, which allows the instrument to be held and directed with more facility. A stilet of silver (*le mandrin*) fills the cavity of the sound, and serves the double purpose of keeping the interior clean, and of preventing the escape of urine, when it is suffered to remain in its cavity. For females, sounds less bent and shorter are employed, called *female sounds*. Most frequently sounds are made of gum-elastic, especially when they are intended to remain sometime in the urethra and bladder. In the divers operations practiced upon the urinary passages, surgeons likewise make use of *solid* sounds, firm and unyielding or flexible to which the names of *bougies* and *catheters* have been given."

In describing *French* operations, it is not astonishing I should call the instruments after the names universally in use in France; but as you are better acquainted with what will answer the generality of American readers than I am, I submit, to your better judgement, the use that may be made of the present communication.

Yours respectfully,

ANTHONY HERMANGE.

*Cincinnati, June,* 1834.

